# Clinical Ticket of the Commercial Hospital of Ohio

**Published:** 1834

**Authors:** 


### IV.—Clinical Ticket of Admission to the Commercial Hospital and Lunatic Asylum of Ohio.

By the charter of this establishment, as enacted in 1821, the professors of the Medical College of Ohio, were made, *ex-officio*, its medical and surgical attendants, with the privilege of introducing the pupils of the college, in the winter season, to witness the treatment of the sick. For this admission, they were at liberty, and, indeed, by the spirit of the law, required to charge; and the moneys thus accruing, were expressly ordered in the same law, to be appropriated to the purchase of books, anatomical preparations, and philosophical apparatus, for the use of the college, and to become the property of the college. Thus an annual revenue was established, by which the *materiel* for instruction was gradually to be procured. This provision was in force from the passage of the act of incorporation, till the year 1826, when at the instance of the Faculty and Trustees it was repealed. Now it might be supposed, that during that period, something had accrued to the literary fund of the college, from the annual sale, in the winter season, of hospital tickets, but nothing could be more distant from the fact. During the whole of that period, not a single cent found its way into the *treasury* of the *college*, but was appropriated by the *professors* to the payment of their *contingent expenses!* Perhaps a more straightforward violation of the plain letter of a statute, could not easily be found. That the *professors* should have been willing to act thus, can be comprehended, as it was for their *personal* interest to do so, but that the courtesy of the Board of Trustees should have been so great, as to permit this continued course of speculation, must seem strange, to those who have had opportunities to observe their general conduct and management.

But this is not the only mode in which the professors have used the hospital for their own personal advantage and advancement. In the vacation, the college had no pupils, and consequently no students were, then, entitled to admission into the hospital. It is in vacation, however, when the number of students in the city is small, that the practice of the hospital is most instructive. But how could they then obtain admission? *In no other way than to become the private pupils of the professors;* who had no legal right, however, to introduce them (seeing that they only had the right to admit the pupils of the *college*) but who, nevertheless, have done so from the beginning down to the present time. They have on the same principle, opposed the establishment of a summer ticket, by the managers of the hospital, although a respectable number of the physicians of the city, several years ago, memorialized the board on that subject. Thus the professors have held out the practice in the hospital, as a *bonus* to young men, about to commence the study of medicine, to become their pupils, instead of the pupils of the other physicians of the city; and then complain that they are surrounded by those who are hostile to their interests! The students of the other cities of the United States, which have hospitals, can study with *whom they please*; and by purchasing a summer ticket, may still enjoy all the benefits of witnessing the practice in those infirmaries; but to enjoy it in Cincinnati, at a hospital established and supported by the state, they must become the *private* pupils of the professors! Every citizen of Ohio, who may wish to place his son to the study of medicine, with any other physician of the city than a professor, is interested in the correction of this abuse.

